# Understanding patient needs and gaps in radiology reports through online discussion forum analysis

**DOI:** 10.1186/s13244-020-00930-2

**Published:** 2021-04-19

**Authors:** Mohammad Alarifi, Timothy Patrick, Abdulrahman Jabour, Min Wu, Jake Luo

**Affiliations:** 1 College of Health Sciences, University of Wisconsin Milwaukee, Milwaukee, WI 53211 USA; 2 College of Applied Medical Sciences, King Saud University, Riyadh, Saudi Arabia; 3 College of Engineering, University of Wisconsin Milwaukee, Milwaukee, WI 53211 USA; 4 Health Informatics Department, Faculty of Public Health and Tropical Medicine at Jazan University, Jazan, Saudi Arabia

**Keywords:** Radiology, Radiology report, Radiology notes, Social media, Online discussion forums

## Abstract

Our objective is to investigate patient needs and understand information gaps in radiology reports using patient questions that were posted on online discussion forums. We leveraged online question and answer platforms to collect questions posted by patients to understand current gaps and patient needs. We retrieved six hundred fifty-nine (659) questions using the following sites: Yahoo Answers, Reddit.com, Quora, and Wiki Answers. The questions retrieved were analyzed and the major themes and topics were identified. The questions retrieved were classified into eight major themes. The themes were related to the following topics: radiology report, safety, price, preparation, procedure, meaning, medical staff, and patient portal. Among the 659 questions, 35.50% were concerned with the radiology report. The most common question topics in the radiology report focused on patient understanding of the radiology report (62 of 234 [26.49%]), image visualization (53 of 234 [22.64%]), and report representation (46 of 234 [19.65%]). We also found that most patients were concerned about understanding the MRI report (32%; *n* = 143) compared with the other imaging modalities (*n* = 434). Using online discussion forums, we discussed major unmet patient needs and information gaps in radiology reports. These issues could be improved to enhance radiology design in the future.

**Supplementary information:**

**Supplementary information** accompanies this paper at 10.1186/s13244-020-00930-2.

## Key points

- A large gap between patients' understanding and current radiology reports.
- Communication sites are an important way to understand patient needs.
- Patients' understanding should always be taken into consideration.
- Providing appropriate reports to understand patients should be a priority.

## Introduction

A radiology report is the official record of medical images that contains the interpretations and images [1]. The main goal of the radiology report is to present the outcomes of the imaging procedure (e.g. X-ray, MRI) of the patients to physicians [2]. Recent studies show that patients want to read their own report or the reports of family members. Oftentimes, they have difficulty understanding the content presented in the reports [3–7]. Many patients are now able to access their radiology reports online [8–10]. This encourages patients to communicate with doctors about their radiology imaging results [10]. A study conducted involving 61,131 patients found that there was a high percentage of patients (51.2%) who reported that they were interested in browsing and reading radiology reports online [8]. This study confirmed patient interest in reading radiology reports. A study of two outpoint groups who had recently undergone MRIs found that most patients were not satisfied with current radiology reports because the reported results were not easy to understand. The same study showed that there is a lack of detail and cited delays in report release as the most important problem with radiology reports. Patients generally preferred to have the option to access more detail in the reports [11].

The involvement of patients in the therapeutic and diagnostic stage has positive benefits [12–15]. Good patient understanding of health reduces the time a doctor must spend explaining treatment steps [16]. In the field of radiology, radiologists and the doctors radiologists refer patients to complain about the lack of time to write reports as well as the time they must use to explain procedures to patients [17]. Giving patients access to their radiology reports provides them with the opportunity to understand the reports prior to meeting with the doctor [4, 18]. Patients access to clear and full radiology reports enables them to share it with other specialists to obtain further explanations, second opinion, or continuous treatments [19, 20]. This can enhance patient’s understanding of the treatment steps, which can helps raise the efficiency of treatment and a better understanding of the health condition can reduce the level of anxiety [21, 22]. Some studies have shown that radiologists fear that patients’ current lack of understanding of the radiology reports could increase anxiety [23, 24]. This concern raises the important issue of the extent of the gap between patient understanding and the current radiology report design. This study will aim to identify the extent of this gap.

**Fig. 1:**
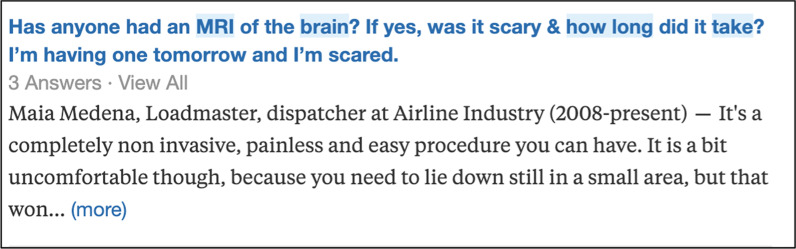
An example of a question from quora.com

The next aim will be to design a more user-friendly radiology report by considering the patient as the primary target of the design. To achieve this objective, we first studied the obstacles involved with submitting the current version of the radiology report to the patient. The results of this study were published in May 2020 [18]. Our current aim is to identify patient desires and priorities for their radiology report by exploring patient questions in online discussion forums (Fig. 1). This study will be the final step before the process of designing a more patient-friendly radiology report begins. Patient comments and questions in online discussion forums were used for a variety of purposes in previous studies [25, 26]. One research study looked at patient concerns about the nature of the healthcare environment based on social media questions. This study collected data from social media sites, including online discussion forums, to find ideas that would help to create a kinder, more reliable healthcare environment [27]. Online discussion forums can be used in the pharmaceutical field to evaluate drugs based on patient questions. For instance, a study used online discussion forums to identify potential candidates for a drug repurposing study and created five potential drug repurposing candidates [28]. This strategy can also be used to understand the medical terminology challenges that patients face. Popular data sources such as Yahoo!Answers, WebMD community, PatientsLikeMe, and Tumblr were studied to understand the language gap between consumers and health practitioners [29, 30].

## Methods

### Data sources and collection

To understand patient needs and gaps in radiology reports, we conducted a scan of four online discussion forums to evaluate the publicly available content addressing patient concerns about their radiology reports. The four sites examined were Yahoo Answers, Reddit.com, Quora, and Wiki Answer (Additional file 1: Appendix A). All questions were in English and not duplicated (Fig. 4). The analysis occurred in the following four steps: determine what websites should be used, collect questions, filter the data, and categorize the questions. To reach more people, the most frequent and recommended platforms from previous studies were used [4–6]. Yahoo Answers is the largest consumer Q&A site and is available in 12 languages, one of which is English. Wiki Answers is another large platform that allows people to ask and answer any question. Quora and Reddit.com are online platforms where patients can ask about and share their own experiences. They are all public websites that allow people from all parts of the world to answer the same question. The platforms allow patients to share their own experiences and improve outcomes.

The search keywords used were selected by the author, a specialist in the radiology field who is familiar with the terms used. The goal was to determine patient needs and gaps in radiology reports. The researchers collected all radiology-related questions as they worked toward their goal. Some procedures that were tracked included radiology modalities such as Magnetic Resonance Radiology (MRI), Nuclear Imaging, Ultrasound, X-Ray, Computer Tomography (CT), Fluoroscopy, and Angiography. Other procedures included radiology services for specific indications like breast cancer or lymphoma. The questions were manually collected using search terms that included the following: radiology reports, radiology modalities, and radiology interpretations.

### Thematic analysis

**Fig. 2:**
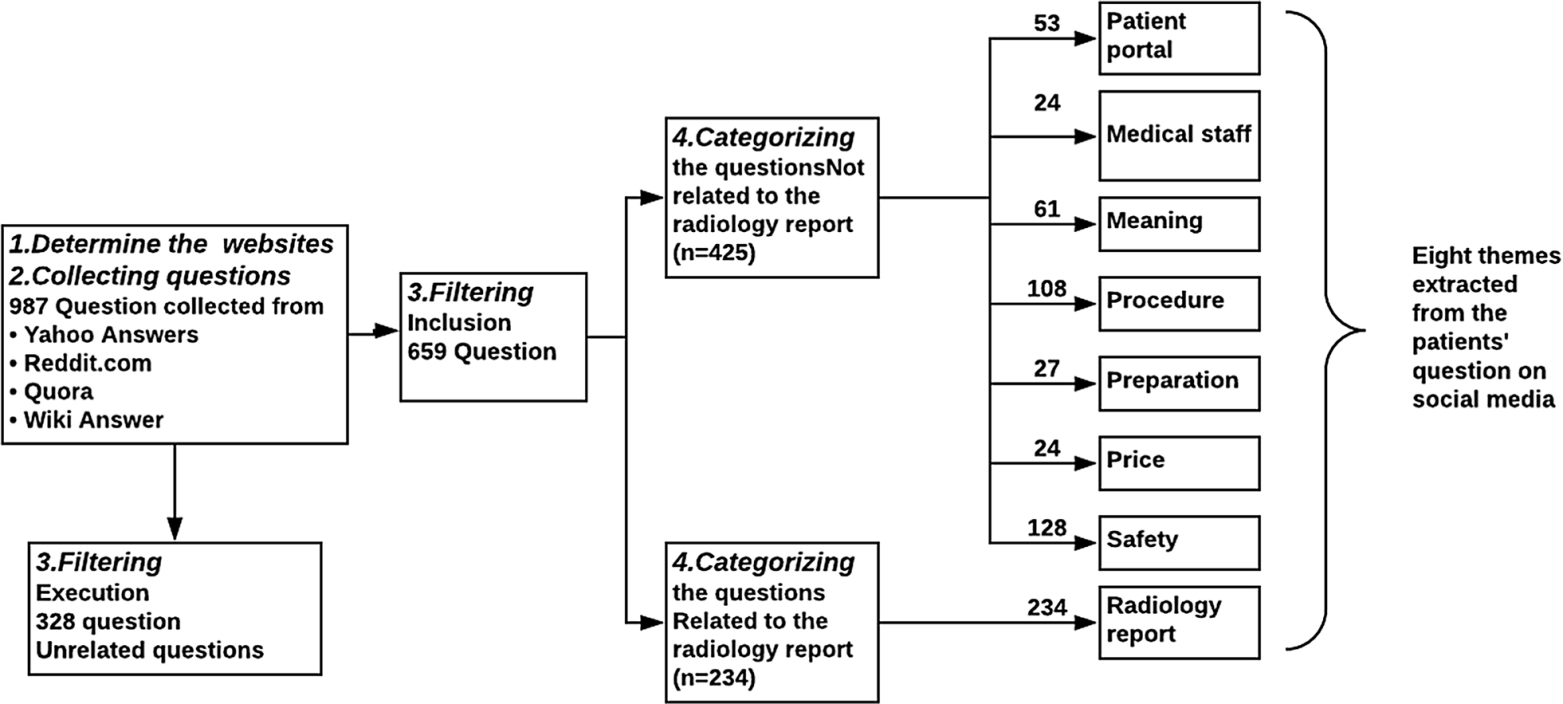
Filtering the questions and creating the eight themes

A total of 987 questions were collected. Of these, 328 questions were discarded because they did not meet the quality criteria which included the following: unrelated to radiology, no clear topic, not in English, and/or contained confidential information. Questions that were added by medical students to answer homework or exam questions were excluded along with any questions that had a patient’s identifying information or full name. We extracted 659 questions from the collected data (Fig. 2).

### Patient-centered approach

**Fig. 3:**
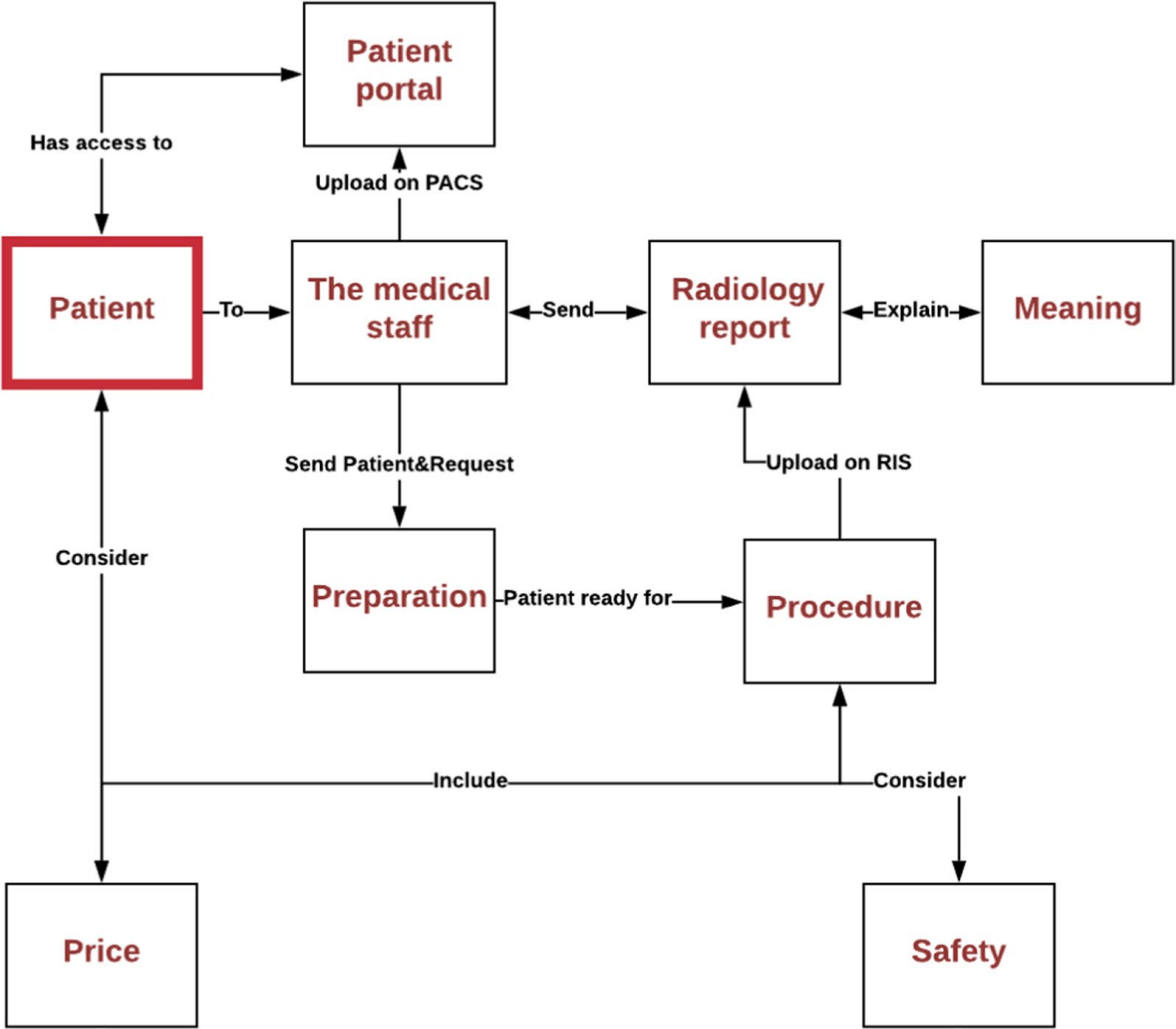
Diagram of the relationship between the 8 themes

Patients are the focus of our research and we searched for the most important themes to understand the radiology report (Fig. 3). The first phase focused on gathering as many questions about radiology as possible from online discussion forums. Based on the review of these forums, we created the eight themes that involved patient concerns regarding the radiology report. The eight themes complement one another to some degree. We also found that patients asked many questions about radiology scan pricing. They wanted to learn about how to access their medical files through the portal. Additionally, some questions showed patient concerns about problems with the portal and the deficiencies of the portal. The questions also inquired about the best doctors based on procedures and test as well as the intricacies of the test itself including those who were responsible for giving instructions and explanations of the scan. The majority of the questions were about safety. The second phase focuses on analysis of the eight themes based on percentage. This allowed us to discover unmet patient needs and information gaps in the radiology reports. We started by categorizing the questions into two main themes, questions related to the radiology report (*n* = 234) and questions not related to the radiology report (*n* = 425). Some questions were not directly related to the radiology report but were generally related to the radiology scan. For thematic analysis, we adopted a grounded theory approach in which themes emerged from the data [31]. We reviewed the questions extracted for topics and themes, then we grouped similar topics and developed a hierarchical code of themes. Extracting topics and themes from questions was conducted independently by two researchers (M.A. and J.L.). The topics were merged if they agreed with one another. If disagreements between the reviewers were identified, they were discussed until a consensus was reached. If no consensus reached, both topics were kept. Eight themes and 19 sub-themes were developed (Table 1). The main eight themes are as follows: the radiology report, patient portal, medical staff, meaning of terms, procedure, preparation, price, safety (Fig. 2). There were six themes that concerned patients: radiology report, safety, price, preparation, procedure, and meaning. The themes and sub-themes were later reviewed and altered by the author, committee reviewers, and the radiologist involved in this study.

## Results

### Summary of the four data sources

See Table 1.

**Table 1:** The main themes that concern patients in radiology

Main themes	Sub-themes	% within the theme	% out of 659
Radiology report (*n* = 234) (35.50%)	Report Representation	19.65	6.98
	Image visualization	22.64	8.04
	Resources	8.54	3.03
	Preference	22.64	8.04
	Understanding	26.49	9.40
Safety (*n* = 128) (19.42%)	Pregnancy	23.43	4.55
	Radiation	29.68	5.76
	Anxiety	32.03	6.22
	Contrast media	14.84	2.88
Procedure (*n* = 108) (16.38%)	Length of procedure	23.15	3.79
	Cognitive questions	45.37	7.43
	Feeling comfortable	31.48	5.15
Meaning (*n* = 61) (9.25%)	Modality types	45.90	4.24
	Test types	54.09	5
Patient portal (*n* = 53) (8.04%)	Technical issues	45.28	3.64
	Features	54.71	4.40
Preparation (*n* = 27) (4.09%)	Preparation	100	4.09
Price (*n* = 23) (3.64%)	Price	100	3.64
The medical staff (*n* = 24) (3.64%)	Responsibility	100	3.64
Price (*n* = 23) (3.64%)	Price	100	3.64

### Analysis of patient question themes

#### Radiology report results

**Fig. 4:**
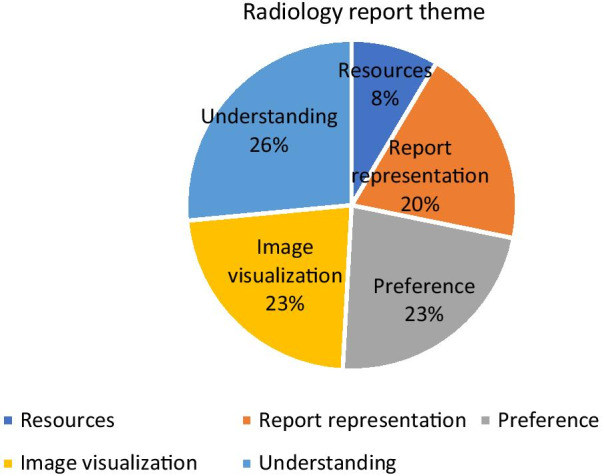
Percentage of questions of sub-themes among in radiology report theme

This theme involves the step after the test and includes radiology images and interpretations. The report results were the major concern for patients with a percentage of 35.50% (*n* = 234) (Table 1). A total of 234 questions were sorted into five sub-themes based on question times. The sub-themes were *report representation (19.65%), resources (8.54%), understanding (26.49%), image visualization (22.64), and preference (22.64%)* (Fig. 4)*.* **Report representation** includes any question about the format of the radiology interpretations, such as font size, font color, unstructured information, information abundance, and confusion about what documents pertain to what information. **Resources** include any external resources such as links and brochures that provide further information to patients about a variety of topics. **Understanding** refers to any question related to issues such as explanations, unclear medical terms, and general confusion about results. **Image visualization** refers to any issues about images, resolution, enhancement, contrast, and color. **Preference** refers to the way that results can be given. These five issues all work to allow patients to contribute to their radiology procedure experience to increase the quality of their diagnostic reports.

#### Safety

These questions refer to concerns involving safety including radiation exposure, medical errors, contraindications, and other negative consequences that could be experienced as a result of the procedures. These issues made up 19.42% (*n* = 128) of questions (Table 1). Questions about the anxiety that can lead patients to cancel their imaging appointments comprised 32.03% of questions. One of the patients asked about travelling by airplane after a nuclear imaging scan. A second major patient concern was about radiation which comprised 29.68% of questions. The other sub-themes were pregnancy and contrast media injections which comprised 23.43% and 14.84% of questions, respectively. An example of a question with this them is "Should all female trauma patients be given a pregnancy test to prevent accidental exposure to radiation?".

#### Procedure processing

This theme involves performing a series of operations during the procedure. We found that 108 of 659 (16.38%) patients were concerned with the steps in the test. Additionally, 45.37% of patients posed cognitive questions. Many questions were about differences in various radiology procedures. Additionally, 23.15% of question involved the length of time that a procedure would take. Patients were less concerned about the length of the procedure than they were about feeling comfortable during the procedure (Table 1).

#### Meaning

We included any question that evidences a lack of basic patient knowledge of the radiology field (9.25%). The questions could involve radiology modality types (45.90%) and test types (54.09%) (Table 1). Modality types and test types are shown as some of the sub-themes in Fig. 4. An example of a question regarding modality and test type is "What is the difference between an open MRI and a closed MRI?" or "What is angiography?".

#### Preparation Patient portal

The patient portal is a secure website that allows patients to access their own medical records. Our findings showed that 8.04% (*n* = 53) were concerned with the patient portal. The concerns were divided into two sub-themes, technical issues (54.28%) and features (54.71%) (Table 1). Technical issues referred to any issue that patients could face such as finding or downloading the report. An example of a technical issue is “I have set up my account, but it isn’t activated” and “My portal has limited options.”

#### Preparation

Preparation includes concerns regarding test preparation including clothing, fasting, and drinking of liquids. The concerns include all radiology types at a percentage of 4.09% (*n* = 27) (Table 1). Many questions were about MRI test preparation. Patients are given MRI instructions for their own safety and these instructions are critical to help patients avoid danger. An example of this is how patients must be free of certain minerals during the examination, especially if the minerals are in the heart valve. It is important to ensure that the patient is free of metal prior to entering the imaging room. This particular requirement has raised issues about the particular examination such as the question of "Why are the instructions of the MRI scan so complicated?".

#### The medical staff

Medical staff is a term that refers to the individuals responsible for preparing the full radiology report, including the image generation process. The term can include radiologists, physicians, and radiology technicians. Our findings show that this theme is asked about with a percentage of 3.64% (*n* = 24) (Table 1). Additionally, 77.77% of questions were about radiologists. As an example of question in this theme is "What is the difference between the physician and the radiologist?"

#### Price

Questions about the price of radiological imaging represented 3.64% (*n* = 24) of questions (Table 1). Many questions were about MRI costs along with questions about what insurance would or would not cover. Despite our findings, patients want to get the best diagnostic imaging even if the cost is high. Many patients have insurance that will cover these costs. There are many potential variables that could increase imaging cost such as the radiology modality and facility capacity. An example of a question that patients asked is “How much does an MRI or CT scan of the head cost?”

## Discussion

In this study, we examined patients’ needs and the gaps related to radiology reports through an investigation that used patients’ responses to questions and posts made on social media websites. To our knowledge, this study is the first to explore patients’ needs and gaps in radiology reports using these sources. Previous studies that have focused on patients’ opinions or challenges related to patients being able to access their full radiology report indicated that patients had an interest in having full access to these reports [4, 18]. A study showed that 51% of patients had a strong desire to obtain their radiology reports without obstacles or challenges [8]. while other studies found that doctors did not mind giving patients their radiology reports; however, doctors feared that patients would not understand the report content properly [23, 24]. Some studies have worked to determine the level of patient satisfaction with current radiology reports [8, 32]. One of these studies showed patients were dissatisfied with the current reports due to the difficulty in understanding the reports [33]. These studies did not address specific patient concerns about radiology reports. One study used patient evaluations posted on yip.com to determine the most important factors related to positive and negative patient perceptions of radiology centers in the United States [34]. This study evaluated the performance of medical radiology centers, but did not address general patient concerns about the overall radiology field [34]. Some studies have focused on the benefits provided by free texting [35, 36] and discussed the positive impact that shortening the time required to write reports can have on doctors and radiologists [37]. This study showed that free texting (creation of unstructured reports) created an output for reports that was not geared toward patients [2, 20]. Proponents of this strategy question the added value of a treatment plan that does not truly involve patients in the understanding of his or her own report [24, 38]. These studies show the gap between patients and the current radiology reports being generated.

The findings of our study were categorized into the following eight themes: radiology report, safety, procedure, meaning, the patient portal, preparation, medical staff, and price. We found that radiology report was the most commonly discussed theme followed by safety and procedure. We also found that the most important concern for patients is the radiology report. We worked to determine the most interesting and concerning topics about the radiology reports and determined that they were most concerned with proper understanding, image visualization, and report representation. A total of 26.49% of the radiology report’s results focused on patient understanding. From our data, patients want to improve their understanding of the report by enhancing image visualization, report representation, resources, and preference. In addition, we found that there is difficulty understanding medical terms, instructions, and the main report issue(s). Also, 20% of the questions suggested that there is a need to improve report representation which involves issues including the report being unstructured, containing too much information, or containing problematic font or color issues. In image visualization, 23% of patients asked many questions to eliminate ambiguity regarding the images shown and to obtain a better understanding of what they were seeing. The need to increase the level of patient understanding is shown in several studies [39, 40]. One study provided resources to patients to allow them to better understand definitions and medical terminology. Of the 185 patients in the study, the majority showed that these additional resources were useful in improving understanding [39].

We also found that some of the findings such as radiation safety were extensively covered in previous studies [41–43]. These studies discussed the protocols followed in protecting patients from radiation and were the second most common element that patients were interested in (19.42%). Patients had other radiation safety concerns including how radiation interacted with pregnancies and how injected materials and anxiety about procedures might factor into treatment. Certain topics such as procedures, meanings, patient portal, medical staff, and pricing were of least concern to patients. Based on past studies, there were still deficiencies in covering the topics that patients found least concerning.

The findings of the study have significant implications for the development of a patient-friendly radiology report that can improve patient understanding. One of the key findings is that patients do not understand radiology reports well and that the reports are negatively impacted by a lack of image visualization and report representation.

### Recommendations

1. Including some of the relevant findings in the report such as blood tests and the patient’s genetic history can be valuable for future decision making
2. Reconsidering the report design to make the report more organized and reviewing the level of language used can be useful
3. Presenting some of the results quantitatively such as dimensions, volumes, Hounsfield numbers, and ADC values in graphs without additional interpretation could aid in understanding
4. The report could include tips and instructions to increase the level of patient satisfaction
5. Future work could occur to determine the value of automating quality control for radiology reports and create a more patient-friendly product

## Conclusion

Patients believe that considering their needs to fill gaps in report representation and image visualization can provide a better understanding of the full radiology report. The new design of the report must consider the following three sub-themes:

1. **Report representation—**this refers to the issues involving font size, colors, unstructured information, too much information, and confusing content
2. **Image visualization—**this refers to issues related to the image itself such as resolution, contrast, enhancement, annotation, and color issue
3. **Understanding—**this refers to questions related to patient understanding including what items require more explanation, unclear medical terms, and general confusion about content

By using online discussion forums, we were able to successfully discover major patient needs and gaps in the current radiology report format. This result shows that it is important to design a consumer-friendly radiology report that focuses on major patient concerns. The design of the report must be universally adopted and applicable to all modality types. Another topic that has been discovered is why patients are more concerned with the MRI report than they are with other radiology reports.

## Supplementary information

**Additional file 1.** Appendix A: Number of questions for each theme among the four online discussion forums.

## Data Availability

The datasets used and/or analyzed during the current study are available from the corresponding author on reasonable request.

## Abbreviations

CT: Computed tomography
MRI: Magnetic resonance imaging
PACS: Picture archiving and communication system

## References

[1] Dunnick NR, Langlotz CP (2008). The radiology report of the future: a summary of the 2007 Intersociety Conference. J Am Coll Radiol.

[2] Olthof A, de Groot JC, Zorgdrager AN, van Ooijen PM (2008). Perception of radiology reporting efficacy by neurologists in general and university hospitals. Clin Radiol.

[3] Yi PH, Golden SK, Harringa JB, Kliewer MA (2019). Readability of lumbar spine MRI reports: will patients understand?. AJR Am J Roentgenol.

[4] Martin-Carreras T, Kahn CE (2018). Coverage and readability of information resources to help patients understand radiology reports. J Am Coll Radiol.

[5] Hayashi D, Guermazi A (2018). Taking a proactive role in patient management of important incidental imaging findings: how can we increase the ‘value’ of diagnostic radiology service and improve quality of patient care?. Jpn J Radiol.

[6] Gutzeit A, Heiland R, Sudarski S (2019). Direct communication between radiologists and patients following imaging examinations Should radiologists rethink their patient care?. Eur Radiol.

[7] Gray BR, Gunderman RB (2019). Constricting the radiologic lexicon: an orwellian errand?. Acad Radiol.

[8] Miles RC, Hippe DS, Elmore JG, Wang CL, Payne TH, Lee CI (2016). Patient access to online radiology reports: frequency and sociodemographic characteristics associated with use. Acad Radiol.

[9] Lee CI, Langlotz CP, Elmore JG (2016). Implications of direct patient online access to radiology reports through patient web portals. J Am Coll Radiol.

[10] Henshaw D, Okawa G, Ching K, Garrido T, Qian H, Tsai J (2015). Access to radiology reports via an online patient portal: experiences of referring physicians and patients. J Am Coll Radiol.

[11] Johnson AJ, Easterling D, Williams LS, Glover S, Frankel RM (2009). Insight from patients for radiologists: improving our reporting systems. J Am Coll Radiol.

[12] Househ M, Borycki E, Kushniruk A (2014). Empowering patients through social media: the benefits and challenges. Health Informatics J.

[13] Bear RA, Stockie S (2014). Patient engagement and patient-centred care in the management of advanced chronic kidney disease and chronic kidney failure. Can J Kidney Health Dis.

[14] Cukor D, Cohen LM, Cope EL (2016). Patient and other stakeholder engagement in patient-centered outcomes research institute funded studies of patients with kidney diseases. Clin J Am Soc Nephrol.

[15] Martinez LS, Carolan K, O’Donnell A, Diaz Y, Freeman ER (2018). Community engagement in patient-centered outcomes research: benefits, barriers, and measurement. J Clin Transl Sci.

[16] Lee K, Kwon H, Lee B (2018). Effect of self-monitoring on long-term patient engagement with mobile health applications. PLoS ONE.

[17] Langer SG (2002). Impact of speech recognition on radiologist productivity. J Digit Imaging.

[18] Alarifi M, Patrick T, Jabour A, Wu M, Luo J (2020). Full radiology report through patient web portal: a literature review. Int J Environ Res Public Health.

[19] Smith JJ, Berlin L (2001). Picture archiving and communication systems (PACS) and the loss of patient examination records. AJR Am J Roentgenol.

[20] Gunderman RB (2018). The true purpose of a radiology report. J Am Coll Radiol.

[21] Carman KL, Dardess P, Maurer M (2013). Patient and family engagement: a framework for understanding the elements and developing interventions and policies. Health Aff.

[22] Jeffreys MC (2017) Treatment adherence and engagement in a transdiagnostic behavioral treatment for pediatric anxiety and depression. UC San Diego

[23] Johnson AJ, Frankel RM, Williams LS, Glover S, Easterling D (2010). Patient access to radiology reports: what do physicians think?. J Am Coll Radiol.

[24] Rosenkrantz AB (2017). Differences in perceptions among radiologists, referring physicians, and patients regarding language for incidental findings reporting. AJR Am J Roentgenol.

[25] Charlie AM, Gao Y, Heller SL (2018). What do patients want to know? Questions and concerns regarding mammography expressed through social media. J Am Coll Radiol.

[26] Lee K, Hoti K, Hughes JD, Emmerton L (2014). Dr Google and the consumer: a qualitative study exploring the navigational needs and online health information-seeking behaviors of consumers with chronic health conditions. J Med Internet Res.

[27] Sarrazin MSV, Cram P, Mazur A, Ward M, Reisinger HS (2014). Patient perspectives of dabigatran: analysis of online discussion forums. Patient.

[28] Rastegar-Mojarad M, Liu H, Nambisan P (2016). Using social media data to identify potential candidates for drug repurposing: a feasibility study. JMIR Res Protoc.

[29] Park MS, He Z, Chen Z, Oh S, Bian J (2016). Consumers’ use of UMLS concepts on social media: diabetes-related textual data analysis in blog and social Q&A sites. JMIR Med Inform.

[30] Gunderman RB, Baskin MH, Brown BP (2015). When a report is both more and less than a report. J Am Coll Radiol.

[31] Glaser BG, Strauss AL (2017). Discovery of grounded theory: strategies for qualitative research.

[32] Short RG, Middleton D, Befera NT, Gondalia R, Tailor TD (2017). Patient-centered radiology reporting: using online crowdsourcing to assess the effectiveness of a web-based interactive radiology report. J Am Coll Radiol.

[33] Gunn AJ, Sahani DV, Bennett SE, Choy G (2013). Recent measures to improve radiology reporting: perspectives from primary care physicians. J Am Coll Radiol.

[34] Doshi AM, Somberg M, Rosenkrantz AB (2016). Factors influencing patients’ perspectives of radiology imaging centers: evaluation using an online social media ratings website. J Am Coll Radiol.

[35] Abramson EL, Patel V, Malhotra S (2012). Physician experiences transitioning between an older versus newer electronic health record for electronic prescribing. Int J Medical Inform.

[36] Tachakra S, Potts D, Idowu A (1990). Evaluation of a computerised system for medical records in an accident and emergency department. Int J Clin Monit Comput.

[37] Al Alawi S, Al Dhaheri A, Al Baloushi D (2014). Physician user satisfaction with an electronic medical records system in primary healthcare centres in Al Ain: a qualitative study. BMJ Open.

[38] Xiong T, McEvoy K, Morton DG, Halligan S, Lilford RJ (2006). Resources and costs associated with incidental extracolonic findings from CT colonogaphy: a study in a symptomatic population. Br J Radiol.

[39] Cook TS, Oh SC, Kahn CE (2017). Patients' use and evaluation of an online system to annotate radiology reports with lay language definitions. Acad Radiol.

[40] Miller P, Gunderman R, Lightburn J, Miller D (2013). Enhancing patients' experiences in radiology: through patient–radiologist interaction. Acad Radiol.

[41] Sidhu M, Strauss KJ, Connolly B (2010). Radiation safety in pediatric interventional radiology. Tech Vasc Interv Radiol.

[42] Engel-Hills P (2006). Radiation protection in medical imaging. Radiography.

[43] Meisinger QC, Stahl CM, Andre MP, Kinney TB, Newton IG (2016). Radiation protection for the fluoroscopy operator and staff. AJR Am J Roentgenol.

