# Supplementary material for: Understanding patient needs and gaps in radiology reports through online discussion forum analysis
Source: Insights Imaging. 2021 Apr 19;12:50. doi: 10.1186/s13244-020-00930-2 (PMC8055745; doi:10.1186/s13244-020-00930-2)
Supplement: Supplementary file 1 — Additional file 1. Appendix A: Number of questions for each theme among the four online discussion forums. [file 13244_2020_930_MOESM1_ESM.docx]

**ELECTRONIC SUPPLEMENTARY MATERIAL**

**Appendix A:**  Number of questions for each theme among the four online discussion forums

A total of 659 questions were identified from four online discussion forums and categorized into eight themes.
